# Five-Month Observation of Persistent Diabetic Macular Edema after Intravitreal Injection of Ozurdex Implant

**DOI:** 10.1155/2014/364143

**Published:** 2014-02-10

**Authors:** Dominik Zalewski, Dorota Raczyńska, Krystyna Raczyńska

**Affiliations:** ^1^Diagnostic and Microsurgery Center of the Eye LENS, 3a Budowlana Street, 10-424 Olsztyn, Poland; ^2^Department of Anesthesiology and Intensive Care Medicine and Department of Ophthalmology, Medical University of Gdańsk, 17 Smoluchowskiego Street, 80-952 Gdańsk, Poland; ^3^Department of Ophthalmology, Medical University of Gdańsk, 17 Smoluchowskiego Street, 80-952 Gdańsk, Poland

## Abstract

*Aims*. This retrospective analysis was aimed at evaluating the effectiveness of treatment of persistent diabetic macular edema with intravitreal injections of 0.7 mg dexamethasone implant Ozurdex. The study comprised three male patients (6 eyes). *Results*. The average thickness of the retina at baseline was 632 **μ**m, the medial BCVA was 0.8 logMAR, and corrected intraocular pressure was 13.7 mmHg. The maximum decrease in mean retinal thickness was observed at four weeks following the treatment and was 365 **μ**m (−267 **μ**m) and visual acuity improved by an average of two lines and was 0.6 logMAR. The largest increase in mean retinal thickness to average of 528 **μ**m (+164 **μ**m) occurred at 16 weeks and the average BCVA was 0.614 lines BCVA logMAR. In one eye, there was a steroid cataract development after the third dose of dexamethasone implant of 0.7 mg. *Conclusions*. The intravitreal dexamethasone implant treatment of patients with persistent diabetic macular edema in whom laser photocoagulation proved to be ineffective and as a result they required a monthly injection of anti-VEGF factors (Ranibizumab, Bevacizumab) may be a good alternative to extending the interval of injections. However, reinjections involve a high risk of developing poststeroid cataracts, which is not without significance in middle-aged patients.

## 1. Introduction

Diabetic macular edema is the leading cause of significant loss of visual acuity in patients with diabetic retinopathy. It results from the damage and increased permeability of macular capillaries in the course of hypoxia, leading to increased levels of antivascular endothelial growth factor (VEGF) and release of inflammatory factors such as cytokines, which result in the loss of endothelial cells and pericytes [1].

The treatment of diabetic macular edema is still difficult and involves a high percentage of failures. The gold standard for laser photocoagulation can indeed maintain and even improve the long-term observation of patients with baseline visual acuity, but in others it is ineffective, and, besides, it leads to reduction in the field of vision, impaired colour perception, and reduced feeling of contrast [2–4]. Used interchangeably, but often in addition to laser, steroids increase the chance of improving visual acuity in patients with DME [3, 5, 6].

Corticosteroids demonstrating strong anti-inflammatory properties reduce the formation of secondary macular edema of various etiologies by reducing capillary permeability, inhibiting fibrin deposition, and retarding the loss of endothelial tight junction proteins. Restricting the migration of leukocytes inhibits the formation of VEGF factor, prostaglandins, and other proinflammatory cytokines [7].

It seems that the route of administration of steroid drugs is crucial for the effectiveness of their action. Orally administered, with the highest concentration in serum, they may cause a significant number of side effects such as Cushing's syndrome, osteoporosis, or exacerbation of diabetes and, at the same time, low levels of steroid concentration in the vitreous. The topical, subconjunctival, and peribulbar method of administration provides a relatively high concentration in the vitreous and in serum; the risk of complications of general administration is similarly high [8, 9].

The use of drugs administered directly into the vitreous can result in achieving the appropriate concentration of the drug directly at the site of the disease with decreasing systemic side effects. One of the most potent anti-inflammatory steroid medications is dexamethasone. Its effect is six times stronger than that of triamcinolone acetonide [10].

Due to the short half-life of dexamethasone in the vitreous, triamcinolone acetate (Kenalog-40, Bristol-Myers Squibb, Princeton, NJ) is widely used in the treatment of secondary macular edema, including diabetic retinopathy, of which lipophilic crystals are deposited in the vitreous for several months. However, this form of triamcinolone acetonide deposit, administered at a dose of 4 mg in a single injection, did not provide a constant level of drug in the vitreous even during the initial period of observation and was associated with side effects such as increased intraocular pressure and steroid cataracts [11, 12].

Introduction in 2009 of the treatment intravitreal implant dexamethasone 0.7 mg (Ozurdex Allergan Inc., Irvine, CA) in the form of a copolymer of lactic acid and glycolic acid—PLGA (Novadur Allergan Inc.)—by the progressive biodegradation made it possible to obtain a comparable concentration in the vitreous chamber for a period of up to 180 days after a single injection.

For as long as that, the drug should provide a better therapeutic effect and reduce the number of intravitreal injections of drugs with a shorter duration of action as well as reduce the risk of adverse effects associated with multiple injections and high concentration immediately after injection [13].

## 2. Methods

This retrospective analysis was to evaluate the effectiveness of treatment of persistent diabetic macular edema with intravitreal injections of 0.7 mg dexamethasone implant Ozurdex.

The study comprised three male patients (6 eyes), aged on average 52 and with average diabetes duration of 13.3 years. In each eye, persistent macular edema occurred for more than six months despite the treatment of grid macular photocoagulation and antivessel growth factor (Ranibizumab) 0.5 mg intravitreal injections. No lens opacities at baseline were observed in any of the patients before treatment. The initial and control studies evaluated the best corrected visual acuity (BCVA logMAR lines), corrected intraocular pressure, and examination of the anterior and posterior segment of the eye with central retinal thickness measurements using optical coherent tomography Spectral Cirrus HD-OCT Zeiss.

Injections were carried out in the operating theatre. Each patient received 0.3% Ciprofloxacin in the eye drops four times a day two days before and four days after the treatment (Figure 3).

## 3. Results

Before the injection of intravitreal implant Ozurdex, each of the six eyes presented significant edema of the retina treated before with grid laser photocoagulation and three intravitreal injections of anti-VEGF factor. The last injection of Ranibizumab 0.5 mg was performed at least 3 months before starting the treatment with Ozurdex. Five eyes indicated with the numbers 4, 5, 6, 7, and 8 received a single injection dose of 0.7 mg Ozurdex, while one eye injection numbered 1, 2, and 3 received three Ozurdex doses at six-month intervals (Table 1).

**Figure 1:**
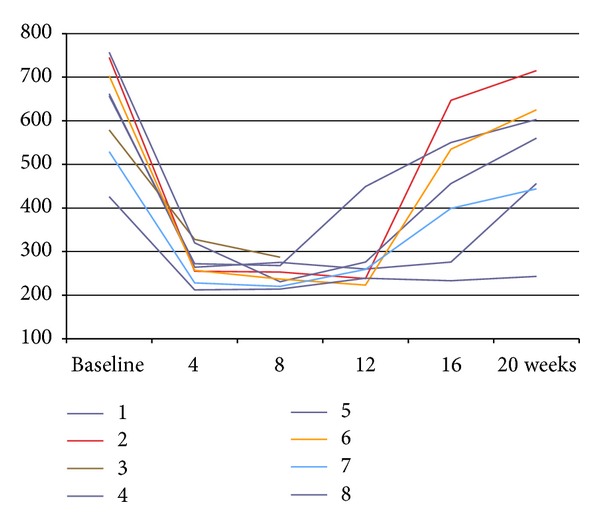
Retinal thickness dependence of the duration of Ozurdex 0.7 mg in the vitreous. Coloured lines refer to the serial numbers of injections from Table 1.

**Figure 2:**
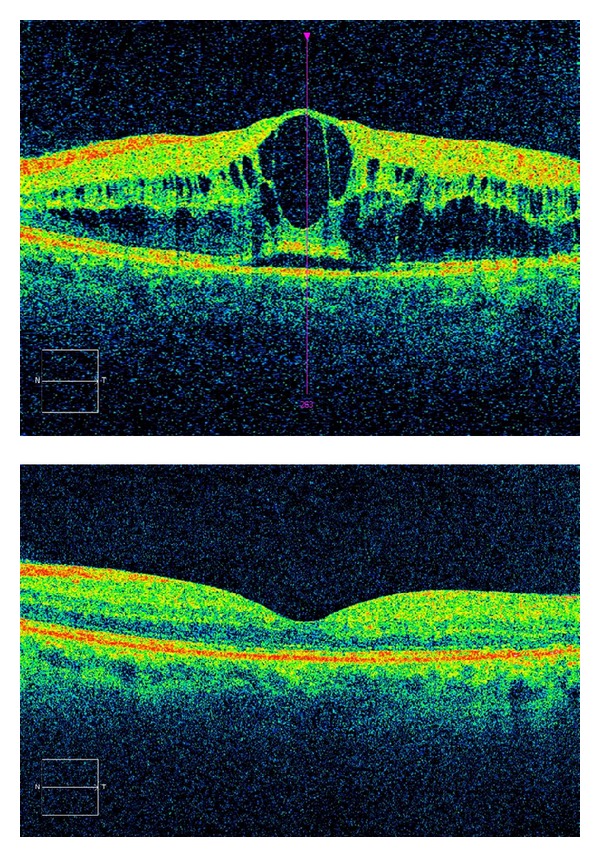
OCT scans: before and one month after intravitreal Ozurdex 0.7 mg injection.

**Figure 3:**
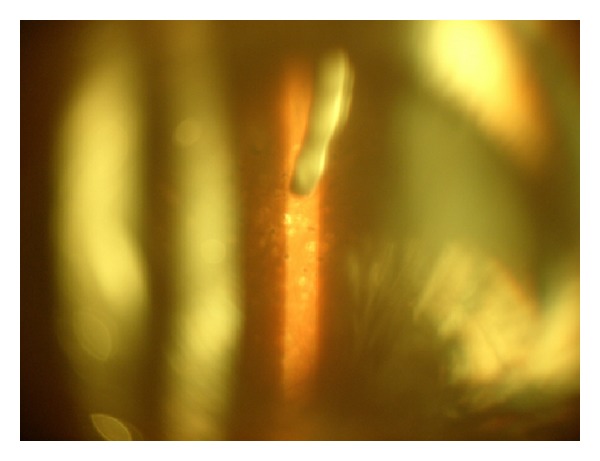
Intravitreal Ozurdex 0.7 mg implant 2 days after injection.

The average thickness of the retina at baseline was 632 *μ*m, the medial BCVA was 0.8 logMAR, and corrected intraocular pressure was 13.7 mmHg. The maximum decrease in mean retinal thickness was observed at four weeks following the treatment and was 365 *μ*m (−267 *μ*m) and visual acuity improved by an average of two lines and was 0.6 logMAR (Figure 2). A slight decrease in retinal thickness to 346.13 microns (−18.87 microns) and mean visual acuity of 0.65 logMAR lines were observed in week 8. At 12 weeks, the average thickness of the retina increased to 381.13 microns (+35 *μ*m) and visual acuity remained stable; however, the results of injection number 3 were excluded because of the occurrence of steroid cataract which significantly reduced visual acuity in the study eye. The largest increase in mean retinal thickness to average 528 *μ*m (+164 microns) occurred at 16 weeks and average BCVA was 0.614 lines BCVA logMAR (Figure 1).

No long-term increased intraocular pressure was observed in this study.

## 4. Discussion

Five-month observation of the patients with chronic diabetic macular edema previously treated with grid photocoagulation and Ranibizumab injections shows that it is not possible to obtain a continuous improvement of the local conditions in visual acuity and decrease in central retinal thickness, which has also been confirmed by other authors [14, 15].

After 4 weeks of injections, all of our patients had a significant decrease in the central retinal thickness and improvement in visual acuity. This condition continued until 12 weeks of observation. In the four-month follow-up, all patients had a gradual increase in the central retinal thickness and their visual acuity after 5 months was similar to that of the pretreatment. In none of the treated patients after this period the central retinal thickness and BCVA logMAR were worse than the baseline value.

In accordance with other literature, the results indicate that the greatest effectiveness of Ozurdex can be seen in the first 4 weeks after administration; it lasts for up to 12 weeks of observation and then gradually decreases [16]. Most likely, this should be associated with the decrease in the vitreous concentrations of dexamethasone released. That confirms the observation that reinjection of Ozurdex after a period of six months produces a similar effect as the first injection.

In our follow-up following administration of 0.7 mg dexamethasone implant, there was no increase in the intraocular pressure requiring medical treatment in any of the eyes.

In one eye, there was a steroid cataract development after the third dose of 0.7 mg dexamethasone implant, which coincides with the observations of other authors [17].

In conclusion, the intravitreal dexamethasone implant treatment of patients with persistent diabetic macular edema in whom laser photocoagulation proved to be ineffective and who, as a result, required a monthly injection of anti-VEGF factors (Ranibizumab, Bevacizumab) may be a good alternative as it allows extending the interval between injections.

## Figures and Tables

**Table 1:** 

Jecti S/NS/N	Baseline		4 weeks		8 weeks		12 weeks		16 weeks		20 weeks	
	CRT	BCVA logMAR	CRT	BCVA logMAR	CRT	BCVA logMAR	CRT	BCVA logMAR	CRT	BCVA logMAR	CRT	BCVA
1	662	0.5	264	0.3	275	0.1	260	0.1	276	0.1	456	0.5
2	745	0.5	255	0.4	253	0.2	238	0.2	647	0.5	715	0.5
3	579	0.5	328	0.5	287	1.2	Posterior capsule cataract occurred					
4	757	1.1	320	0.5	231	0.5	276	0.5	456	0.7	560	1.0
5	657	1.0	272	0.5	268	1.0	449	0.7	550	1.0	603	1.0
6	703	1.2	257	1.1	237	1.0	223	1.0	535	1.0	625	1.1
7	529	1.1	228	1.0	220	1.0	259	0.5	399	0.7	444	1.0
8	426	0.5	212	0.5	214	0.2	239	0.3	233	0.3	243	0.4

BCVA: best corrected visual acuity; CRT: central retinal thickness in *μ*m; S/N: serial number of injection.
